# Outbreaks of *Kingella kingae* Infections in Daycare Facilities

**DOI:** 10.3201/eid2005.131633

**Published:** 2014-05

**Authors:** Pablo Yagupsky

**Affiliations:** Soroka University Medical Center, Ben-Gurion University of the Negev, Beer-Sheva, Israel

**Keywords:** *Kingella kingae*, bacteria, bacteremia, daycare facilities, invasive disease, carriage, transmission, epidemiology, antibiotic prophylaxis, antibiotics, antibacterial drugs, United States, France, Israel

## Abstract

During the past decade, transmission of the bacterium *Kingella kingae* has caused clusters of serious infections, including osteomyelitis, septic arthritis, bacteremia, endocarditis, and meningitis, among children in daycare centers in the United States, France, and Israel. These events have been characterized by high attack rates of disease and prevalence of the invasive strain among asymptomatic classmates of the respective index patients, suggesting that the causative organisms benefitted from enhanced colonization fitness, high transmissibility, and high virulence. After prophylactic antibacterial drugs were administered to close contacts of infected children, no further cases of disease were detected in the facilities, although test results showed that some children still carried the bacterium. Increased awareness of this public health problem and use of improved culture methods and sensitive nucleic acid amplification assays for detecting infected children and respiratory carriers are needed to identify and adequately investigate outbreaks of *K. kingae* disease.

During the past 3 decades, Western countries have reported a rising number of mothers entering the workforce and, consequently, a growing number of children receiving care outside the home (*1*). This trend has substantial public health consequences because the incidence of infectious diseases in general, and of those caused by respiratory pathogens in particular, has substantially increased among daycare center attendees (*1*,*2*). These organisms are usually spread within daycare centers by child-to-child transmission; they colonize the upper respiratory tract surfaces, from which they can disseminate to other attendees. From the upper airways, pathogens may invade adjacent structures such as the lungs, middle ear, or nasal sinuses, and may penetrate into the bloodstream, causing invasive diseases (*1*). Most bacterial pathogens responsible for such infections are enclosed by polysaccharide capsules that protect them from phagocytosis and complement-mediated killing, ensuring their persistence on the respiratory mucosae and survival in the bloodstream and deep body tissues (*3*). Maturation of the T-cell independent arm of the immune system in humans is delayed until the age of 2–4 years; thus, young children are prone to colonization and infection by encapsulated bacteria (*3*).

Besides the microorganisms’ virulence and the hosts’ age-related immunologic immaturity, many other factors contribute to the enhanced colonization, transmission, and illness rates observed among children in daycare centers, including the number of children present, the degree of crowding, efficacy of ventilation, time spent in day care, length of time from enrollment, frequency of enrollment, age group mixing, and occurrence of seasonal viral infections (*1*,*4*–*6*). Because of age stratification, child-care groups comprise attendees of approximately the same age who have similar degrees of immunologic immaturity and susceptibility to infectious agents. This epidemiologic setting substantially differs from that of large families in that the latter include children of different ages and therefore, at any given time, only a fraction of siblings belong to the age group at enhanced risk for bacterial colonization and invasion, which limits the chances to acquire and transmit the organism (*7*). In daycare centers, respiratory organisms spread easily through large droplet transmission among young children with poor hygienic habits who share toys contaminated with respiratory secretions or saliva. Under these circumstances, introduction of a virulent bacterium in a crowded daycare facility attended by immunologically naïve children may result in prompt dissemination of the organism and initiate outbreaks of disease such as those caused by pneumococci, *Haemophilus influenzae* type b, or *Neisseria meningitidis* (*1*,*2*,*6*).

## *Kingella kingae*: An Emerging Pathogen in Young Children

Because of the improved culture methods (*8*,*9*) and sensitive nucleic acid amplification assays (NAAAs) (*10*–*14*) developed in recent years, *Kingella kingae*, a gram-negative coccobacillus of the *Neisseriaceae* family, is increasingly recognized as an invasive pathogen of early childhood. The organism is a frequent source of childhood bacteremia and the most common agent of skeletal system infections in children 6 months–3 years of age; it is also a cause of bacterial endocarditis in children and adults (*8*–*16*). Because of the fastidious nature of *K. kingae*, many illnesses caused by this bacterium are, probably, overlooked. Although most cases of invasive *K. kingae* infections are sporadic, clusters of invasive disease have been detected among attendees of daycare centers in Israel, Europe, and the United States. 

### Misdiagnosis

The bacteriologic identification of *K. kingae* relies on the following: typical Gram stain results, showing pairs or short chains of plump, gram-negative bacilli with tapered ends; β-hemolysis; pitting of the agar surface; failure to grow on MacConkey medium; weak oxidase activity and a negative catalase reaction; production of acid from glucose; and, with rare exceptions, production of acid maltose (*8*,*9*). However, *K. kingae* tends to retain crystal violet dye and, therefore, it may appear to be gram-positive, and laboratories unfamiliar with its cultural and staining features may misidentify the bacterium altogether or dismiss invasive isolates as culture contaminants. Identification of *K. kingae*, however, is not difficult, and many commercial instruments and technologies such as VITEK 2 (bioMérieux, Marcy-l’Etoile, France), matrix-assisted laser desorption ionization-time of flight mass spectrometry (MALDI-TOF), or 16S rDNA gene sequencing, correctly identify the organism.

### Characteristics and Manifestations

Recent studies have demonstrated the presence of a polysaccharide capsule on the surface of *K. kingae* that exhibits between-strains chemical variation and, most probably, antigenic variation (*17*,*18*). These characteristics may assist *K. kingae* in evading the immune response by facilitating the successive colonization of the host by strains harboring different capsular types. The polysaccharide capsule probably enables mucosal colonization, the survival of the organism in the bloodstream, and invasion of deep body sites (*17*,*18*). In addition, all *K. kingae* strains produce and secrete a potent repeats-in-toxin (RTX) that exhibits a wide range of cytotoxic activity and is particularly deleterious to macrophage-like cells, leukocytes, and synovial cells and, to a lesser degree, to respiratory epithelial cells that improve the organism’s chances of surviving in the host and of invading skeletal tissues (*12*,*19*).

Similar to meningococci, *K. kingae* is carried on the oropharyngeal epithelium (*20*,*21*), and the colonized mucosa is the portal of entry of the organism to the bloodstream from which it may disseminate to 3 areas for which the bacterium shows particular tropism: joints, bones, or the endocardium (*22*,*23*). Damage to the upper respiratory surfaces by previous or concurrent viral infections or stomatitis appears to facilitate bloodstream invasion by *K. kingae* (*16*).

*K. kingae* isolates show remarkable genomic diversity and, to date, 37 multilocus sequence typing (MLST) and 74 pulsed field gel electrophoresis (PFGE) clones have been identified (*24*–*26*). Carried *K. kingae* organisms differ in their invasive capabilities (*27*), and simultaneous carriage of ≥1 genotype is unusual (*28*). Whereas some strains, which are frequently isolated from healthy carriers, are seldom if ever detected in patients with clinical disease, others are rarely carried asymptomatically, but are responsible for a high proportion of invasive disease (*24*,*27*,*28*). However, a few strains appear to possess an optimal balance between transmissibility and invasiveness and are common among healthy carriers and among infected patients (*24*,*27*,*28*). A recent study has found that certain virulent *K. kingae* clones, characterized by a distinct combination of PFGE and MLST profiles, are substantially associated with bacteremia with no focal infection, skeletal system invasion, or endocarditis, which suggests biological specialization for invading specific host tissues (*25*).

Most young children in whom an invasive *K. kingae* disease developed have been otherwise healthy. In contrast, children >4 years of age and adults who become infected frequently have underlying conditions such as congenital heart diseases, chronic renal failure, or a variety of primary immunodeficiencies (*16*).

The prevalence rate in healthy children during the second year of life ranges between 10% and 12% (*7*,*28*), which coincides with the peak attack rate of invasive infections (*16*). The colonization rate drops substantially in older children and adults (*7*,*28*,*29*). Pharyngeal carriage of *K. kingae* and occurrence of disease before a child is 6 months of age are exceptions, indicating that maternal immunity and limited social contact provide protection (*7*,*16*,*30*).

### Diagnosis of *K. kingae* Infections

The clinical features of invasive *K. kingae* infections (other than endocarditis) are usually mild, and diagnosis requires a high level of suspicion by clinicians. Many patients with *K. kingae*–associated joint or bone disease are afebrile and blood leukocyte counts, C-reactive protein levels, and erythrocyte sedimentation rates are frequently normal (*16*,*31*,*32*). Although bacteremia with no focus is the second most frequent manifestation of invasive *K. kingae* infections, the condition is probably unsuspected and the diagnosis is likely missed in a large number of cases. The current guidelines for managing illness in young, febrile children with no apparent source of infection, which use body temperature and leukocyte count as criteria for obtaining blood cultures (*33*), are not sensitive enough for detecting occult *K. kingae* bacteremia because many infected children have a low-grade fever and may or may not have leukocytosis.

Detection of *K. kingae* infections by culture is highly dependent on the use of adequate laboratory techniques. The recovery of *K. kingae* from synovial fluid and bone exudates seeded onto routine solid culture media is suboptimal (*8*). The yield of cultures can be substantially improved by inoculating clinical specimens into aerobic blood culture vials (BCVs) from a variety of commercial systems (*8*). Attempts to isolate the organism from synovial fluid or bone exudates on routine solid media succeeded in 2 of 25 patients, whereas inoculation of these specimens into aerobic BACTEC (Becton Dickinson, Cockeysville, MD, USA) BCVs yielded the organism in all cases after a median incubation of 4 days (*8*). When specimens from BCVs determined to be positive by the automated blood culture instrument were subcultured onto a blood–agar plate of trypticase soy agar with 5% sheep blood hemoglobin or chocolate agar, *K. kingae* grew readily, indicating that routine solid media are able to support the nutritional requirements of the organism. This observation suggests that skeletal system exudates exert a detrimental effect on this fastidious bacterium, and dilution of purulent material in a large volume of broth decreases the concentration of inhibitory factors, improving its recovery (*8*). In studies conducted in Israel and France in which BCVs were routinely inoculated with synovial fluid aspirates from young children who had arthritis, *K. kingae* was isolated in 48% of the patients with culture-proven disease (*8*,*9*). Conversely, when BCVs are not used, many *K. kingae* infections will be overlooked and labeled as culture-negative septic arthritis (*34*).

In recent years, development of NAAAs has further improved the diagnosis of *K. kingae* from skeletal system exudates. Use of this novel technique facilitates detection of difficult-to-culture organisms, enables bacteriologic diagnosis in patients already treated with antibacterial drugs, reduces time to detection, and facilitates precise identification of unusual species (*10*–*14*). The procedure consists of extraction of bacterial DNA from the synovial fluid sample, a PCR amplification step in which primers target the *16S rDNA*, the *23S rDNA*, or the *rpo* genes that are ubiquitous to all bacteria, then sequencing of the amplicon, and comparison of results with those kept in a broad database curator (such as GenBank) to enable precise species identification. Alternately, the specimen may be subjected to amplification by PCR by using species-specific primers that recognize the most plausible pathogens. Use of NAAAs has confirmed that in countries where this topic has been studied, *K. kingae* is the most common etiologic agent of septic arthritis in children <3 years of age. NAAA use detected the presence of *K. kingae*–specific DNA sequences, including in cases in which seeding of synovial fluid specimens into BCVs failed to recover the organism and shortened the time required to detect and identify the bacterium from 3–4 days to <24 hours (*10*–*14*). It was, then, natural that this sensitive approach was consequently adopted to study respiratory colonization by *K. kingae* and its connection to invasive disease. 

In an outbreak investigated by Bidet et al., the cause of the skeletal infections was determined by sequential use of real-time PCR targeting the *K. kingae*–specific toxin *rtx*A and the *cpn*60 genes (*35*). The same method was used to investigate the prevalence of *K. kingae* among attendees of the index daycare facility. The organism was recovered by culture in 6 asymptomatic carriers, whereas the NAAs detected 5 additional carriers (*35*). MLST and sequencing of the *rtx*A amplicon were performed directly on the joint fluid sample from the child who had arthritis and on the recovered pharyngeal isolates. All carriage isolates and the arthritis strain belonged to MLST 25 and shared *rtx*A allele 1, which is among the most common genotypes involved in joint and bone infections in France (*26*,*35*). It should be pointed out that, despite the increased sensitivity of NAAAs, when evaluating the efficacy of prophylactic antibacterial drug administration for eradicating *K. kingae* from colonized children, cultures have the obvious advantage of detecting living bacteria, whereas the viability of *K. kingae* organisms in PCR-positive/culture-negative specimens is questionable.

In addition detecting invasive *K. kingae* disease in patients, searching for asymptomatic but colonized children is crucial in the investigation of an outbreak in a daycare facility to assess the full extent of the contagion, identify attendees at risk for clinical disease, and evaluate the effects of prophylactic antibacterial drugs. Because of the high density of the resident bacterial flora and the relatively slow growth of *K. kingae*, detecting the organism in pharyngeal cultures is difficult. A differential and selective medium consisting of blood agar with 2 mg/mL of vancomycin (BAV medium) added has been developed to improve recovery of *K. kingae* from respiratory cultures (*36*). This formulation facilitates recognition of β-hemolytic *K. kingae* colonies by inhibiting growth of competitive gram-positive bacteria. In a blinded evaluation, the BAV medium detected 43 (97.7%) of 44 pharyngeal cultures positive for the organism; 10 (22.7%) positive cultures were identified on routine blood-agar plates (p<0.001) (*36*). If the original BAV formulation (*36*) or a similar medium (*23*) had not been used, and a chocolate-based agar substituted (in which the faint ring of hemolysis surrounding *K. kingae* colonies could not be recognized), carriers of the organism might not have been detected among 27 asymptomatic attendees of a daycare facility where a cluster of invasive disease occurred (*37*).

## Daycare Centers as Reservoirs of Invasive *K. kingae* Disease

The *K. kingae* colonization rate is substantially enhanced among children in daycare centers. In an 11–month longitudinal study, 35 (72.9%) of 48 daycare center attendees carried the organism at least once and an average of 27.5% of the children were colonized at any given time (*20*). Molecular typing of isolates from asymptomatic colonized attendees showed genotypic similarities, indicating person-to-person transmission of the organism in the facility (*21*). Two *K. kingae* strains represented 28.0% and 46.0% of all isolates, demonstrating that some strains are particularly successful in colonizing mucosa. Children harbored the same strain continuously or intermittently for weeks or months, and then it was replaced by a new strain, showing that carriage is a dynamic process in which there is frequent turnover of colonizing organisms, as observed for other respiratory pathogens (*21*,*28*). Despite the high prevalence of the organism in the daycare center, an invasive *K. kingae* infection did not develop in any of the attendees in the course of the follow-up period.

The link between out-of-home child care and *K. kingae* carriage was recently confirmed in a study conducted among 1,277 children <5 years of age who were referred to a pediatric emergency department (*7*). Daycare attendance was strongly associated with *K. kingae* carriage after controlling for other variables (odds ratio 9.66 [95% CI 2.99–31.15], p<0.001) (*7*). Surveillance studies not only have showed that *K. kingae* organisms colonizing attendees of a given daycare center are frequently identical, but have also demonstrated that carried strains differ between facilities located close together, indicating that each daycare center is like an independent epidemiologic unit (*35*,*37*–*39*).

Considering these findings, it is not surprising that clusters of proven and presumptive cases of invasive *K. kingae* disease have been detected in daycare centers in France (*35*), the United States (*37*,*38*), and Israel (*39*), including 2 recent and still unreported outbreaks (P. Yagupsky, unpub. data) (Table 1). A presumptive case was defined as bacteriologically unconfirmed invasive disease, consistent with the clinical features of *K. kingae* infections, among daycare center attendees 6 months–3 years of age, within 1 month of an infection confirmed by culture and/or NAAAs. These events have been characterized by the simultaneous or consecutive occurrence of multiple cases of disease that included the entire clinical spectrum of *K. kingae* infections (septic arthritis, osteomyelitis, bacteremia, spondylodiscitis, cellulitis, and fatal meningitis complicating endocarditis). The 3 patients in the 2005 cluster in Israel (*39*) and 4 of the 5 patients reported in France (*35*) had bone infections, supporting the concept that certain *K. kingae* clones exhibit specific tissue tropism (*25*).

**Table 1:** Demographic and clinical features of 6 clusters of invasive *Kingella kingae* infections in daycare centers in the United States, Israel, and France*

Reference	Year	Country	Attack rate (%)	No. cases confirmed by		No. presump. cases	Patient age range, mo	Outbreak duration, d	Clinical syndromes						
				Culture	NAAA				SA	OM	SD	CE	OB	EN	MN
(*38*)	2003	United States	3/21 (14.3)	2	ND	1	17–21	<14	2†	2†	–	–	–	–	–
(*39*)	2005	Israel	3/14 (21.4)	1	ND	2	8–12	15	–	3	–	–	–	–	–
(*37*)	2007	United States	3/14 (21.4)	2	ND	1	11–25	11	–	–	1	1	–	1‡	1‡
(*35*)	2011	France	5/24 (20.8)	0	1	4	10–16	30	1	4	–	–	–	–	–
P. Yagupsky, unpub. data	2012	Israel	2/36 (5.6)	1	ND	1	10–16	7	1	–	–	–	1	–	–
P. Yagupsky, unpub. data	2013	Israel	2/13 (15.4)	2	ND	0	12	7	2	–	–	–	–	–	–
Total			18/122 (14.8)	8	1	9	8–25	7–30	6	9	1	1	1	1	1

*NAAA, nucleic acid amplification assay; presump., presumptive; SA, septic arthritis; OM, osteomyelitis; SD, spondylodiscitis; CE, cellulitis; OB, occult bacteremia; EN, endocarditis; MN, meningitis.; ND, not done; – indicates that no patients manifested this syndrome. †One child had osteomyelitis of the femur and septic arthritis of the contiguous hip joint.  ‡Meningitis developed in 1 child who had endocarditis.

Three of the 6 *K. kingae* illness outbreaks in daycare centers reported during 2003–2013 were detected in Israel, a small country with a population of 8,000,000 inhabitants (*35**,**37**–**39*; P. Yagupsky, unpub. data). Although Israel’s relatively high annual birthrate compared with Western countries (18.7 per 1,000 population, Israel Central Bureau of Statistics, vs. 10.9 per 1,000 in the European Union, Demography Report 2010, Eurostat), and the widespread and early daycare center attendance could partially account for this observation, it seems plausible that similar events occur worldwide but are frequently overlooked.

The epidemiologic investigation of these outbreaks revealed that the *K. kingae* colonization rate among asymptomatic attendees to the daycare centers where clinical cases were detected was unusually high (up to 52.9% in a United States cluster, as determined by culture [*38*], and 68.8% in France, as demonstrated by a sensitive NAAA [*35*]) (Table 2), and all pharyngeal isolates detected in the classrooms where disease occurred were genotypically identical and indistinguishable from the patients’ clinical isolates. The age of the colonized or infected daycare center attendees coincided with the age of increased susceptibility to *K. kingae* carriage and disease (*7*,*15*,*16*,*28*); the organism was detected in the pharyngeal culture of only1 of the caregivers, supporting the hypothesis that healthy adults rarely carry *K. kingae* (*29*).

**Table 2:** Antibacterial drug prophylaxis administered and its effect on colonization rates and secondary invasive disease after *Kingella kingae* infections in daycare centers, United States, Israel, and France

Reference	Initial carriage rate among healthy attendees (%)	Antibacterial drug prophylaxis					Interval between cultures, d	Post-prophylaxis carriage rate (%)	Post-prophylaxis new cases
		Rifampin			Amoxicillin				
		Dosage, 10 mg/kg 2×/d	Duration, d		Dosage, 40 mg/kg 2×/d	Duration, d			
(*38*)	9/17 (52.9)	Yes	2		No	NA	10–14	4/17 (23.5)	0/17
(*39*)	4/11 (36.4)	Yes	2		Yes	4	2	0/10	0/11
(*37*)	0/27	Yes	2		Yes	2	NA	ND	0/27
(*35*)	11/16 (68.8)†	Yes	2		No	NA	15	11/16† (68.8)	0/19
P. Yagupsky, unpub. data	4/36 (11.1)	Yes	2		Yes	4	10	2/36 (5.6)	0/36
P. Yagupsky, unpub. data	5/11 (45.4)	Yes	2		Yes	4	12	0/11	0/11
Total	33/118 (28.0)	NA						17/90 (18.9)	0/121

*ND, not done; NA, not applicable. †As determined by a nucleic acid amplification assay. In all other cases carriage was established by culture on selective media.

Despite a background carriage rate as high as 5%–12%, the incidence of invasive *K. kingae* infections reported in Israel was 9.4 per 100,000 children <5 years of age per year only (*16*), and the calculated annual risk of developing *K. kingae* osteomyelitis or septic arthritis for young carriers in Switzerland was <1% (*40*). When data from the 6 clusters of invasive disease detected in daycare centers are pooled, a documented or presumptive *K. kingae* infection developed in 1 in 7 classmates within a 1-month period (*35*,*37*–*39*), indicating that the outbreak strains combined enhanced colonization fitness, high transmissibility, and remarkable virulence.

The strain responsible for the cluster of osteomyelitis detected in Israel in 2005 (*39*) belonged to PFGE clone K and MLST-6 that ranked second among strains carried in southern Israel by healthy Jewish children (*20*), and was responsible for the excess of *K. kingae* illness observed in the Jewish population of the region over the previous 2 decades, causing 41.7% of all invasive strains isolated in this ethnic group (*27*). The clone that caused an outbreak in daycare centers in North Carolina, USA (*37*), represented 11.3% of all organisms carried by healthy children in southern Israel (*24*) and 11.6% of all isolates from patients in Israel who had invasive infections (*27*). The international distribution of these clones indicates that the clusters of disease are frequently caused by highly successful strains that exhibit enhanced capability for local and long-range dissemination (*26*).

## Management of *K. kingae* Outbreaks

To prevent further cases of disease and eradicate the invasive strain, prophylactic antibacterial drugs have been administered to children attending the facilities where clusters of *K. kingae* were detected. Rifampin was chosen because *K. kingae* is especially susceptible to that antibacterial drug (*35*). Rifampin is secreted in saliva and reaches high concentrations in the upper respiratory mucosa and has shown efficacy in the eradication of colonization by *N. meningitidis* and *H. influenzae* type b, and in disease prevention in daycare centers (*2*). However, because only partial success was achieved with this drug in the Minnesota cluster (*38*), high-dose amoxicillin was added to the regimen in 2 more recent outbreaks (*37*,*39*). Following administration of antibacterial drugs, a respiratory carriage of the organism decreased, but complete eradication occurred only in the Durham, North Carolina daycare center (*37*), and new colonization of several attendees by the original strain was later observed (*35*,*38*,*39*). Persistence of the organism in the facility was not caused by bacterial resistance to the administered antibacterial drugs, however (*35*,*37*,*39*). Similar observations have been made in outbreaks in daycare centers caused by *H. influenzae* type b and pneumococci (*2*). Poor compliance or failure to administer prophylactic antibacterial drugs to family contacts could have resulted in incomplete suppression of the reservoir and recurrent dissemination of the strain in the facility (*2*).

Because antibacterial drugs have been relatively ineffective in eradicating *K. kingae* carriage, the need for antibacterial drug prophylaxis in the setting of a cluster of invasive disease is being disputed (*40*). Notably, however, after administration of antibacterial drugs to the asymptomatic children, no further cases of disease were detected in the affected daycare centers (*35*,*37*–*39*), even when a few children continue to carry the invasive strain. Reducing the bacterial density among colonized children by antibacterial drug administration or an effective immune response induced by prolonged mucosal carriage may have been sufficient to prevent new cases of infection. Improved hygiene and institution of other infection control measures could also have limited further transmission of the organism. The Figure depicts an algorithm aimed to guide the investigation and management of clusters of invasive *K. kingae* in daycare facilities, including areas of controversy.

**Figure:**
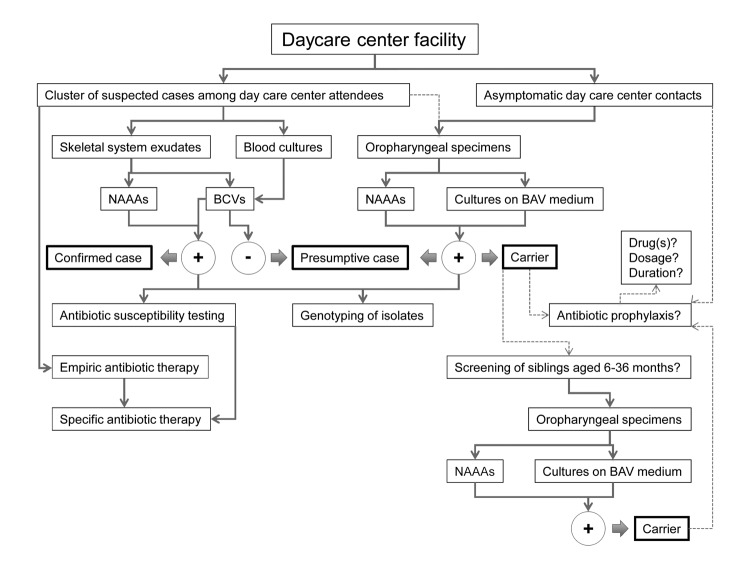
Suggested algorithm for the investigation and management of clusters of invasive *Kingella kingae* infections in daycare centers. Bold boxes indicate case type; dashed lines indicate controversial area. NAAAs, nucleic acid amplification assays; BCVs, blood culture vials; BAV, blood-agar-vancomycin medium.

## Conclusions

In recent years, clusters of invasive *K. kingae* infections among attendees of daycare centers have been reported, although because of the low rate of testing for this pathogen, many events are probably overlooked. Detection of these events requires a high level of clinical suspicion, use of sensitive culture techniques and NAAAs, and familiarity of the clinical microbiology laboratory with the identification of this elusive pathogen. The same improved detection methods should be employed for the thorough investigation of these clusters, and recovered *K. kingae* isolates should be genotyped and compared. Many issues remain unsettled, including whether antibacterial drugs should be administered prophylactically to daycare center contacts of an index case-patient, to young siblings of clinical case-patients, and to carriers, or whether antibacterial drugs should be offered to confirmed carriers only, or limited to children with disease. If administration of antibacterial drug prophylaxis is decided on, the preferred drug regimen will have to be determined. Whether an epidemiologic investigation should be carried out in daycare centers after detection of a single case of disease also remains to be determined.
